# On-demand droplet formation at a T-junction: modelling and validation

**DOI:** 10.1038/s41378-025-00950-2

**Published:** 2025-05-19

**Authors:** Hongyu Zhao, William Mills, Andrew Glidle, Peng Liang, Bei Li, Jonathan M. Cooper, Huabing Yin

**Affiliations:** 1 Division of Biomedical Engineering, James Watt School of Engineering, University of Glasgow, Glasgow, G12 8LT Scotland UK; 2 Institute for Multiscale Thermofluids, School of Engineering, the University of Edinburgh, Edinburgh, EH9 3BF Scotland UK; 3 HOOKE Instruments Ltd., NO. 77 Yingkou Road, Changchun, 130033 Jilin, China; 4 Changchun Institute of Optics, Fine Mechanics and Physics, Chinese Academy of Sciences, Changchun, 130033 Jilin, China

**Keywords:** Engineering, Physics

## Abstract

Droplet microfluidics have found increasing applications across many fields. While droplet generation at a T-junction is a common method, its reliance on trial-and-error operation imposes undesirable constraints on its performance and applicability. In this study, we demonstrate a simple method for on-demand droplet formation at a T-junction with precise temporal control over individual droplet formation. Based on experimental observations, we also develop a physical model to describe the relationships among pressures, droplet generation, device geometry, and interfacial properties. Experimental validation demonstrates excellent performance of the model in predicting the pressure thresholds for switching droplet generation on and off. To address parameter uncertainties arising from real-world complexities, we show that monitoring droplet generation frequency provides a rapid, in situ approach for optimising experimental conditions. Our findings offer valuable guidelines for the design and automation of robust droplet-on-demand microfluidic systems, which can be readily implemented in conventional laboratories for a broad range of applications.

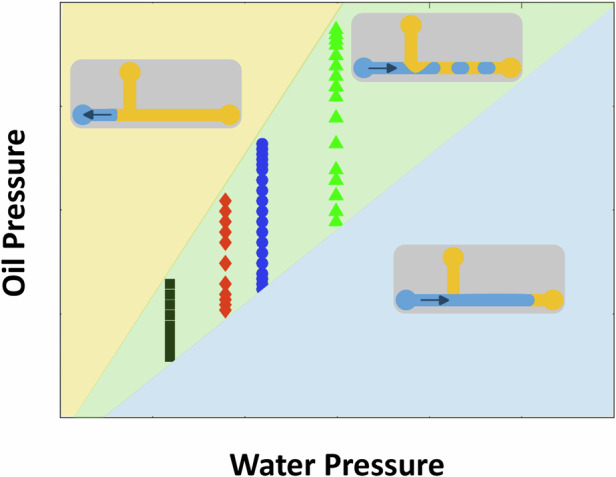

## Introduction

Droplet microfluidics has found increasing applications across many fields since droplets provide an isolated microenvironment, enabling numerous experiments under different conditions, such as cell culture and sorting^1–3^, bioassays^4,5^, and microreactors^6,7^. Typically, droplets are generated through both passive and active methods^8–10^. Passive approaches use remote pressure sources to deliver two immiscible flows to microfluidic junctions (e.g., flow-focusing or T-junctions), generating streams of droplets. These methods offer high throughput but have limited control over droplet formation and individual droplet content. For instance, the encapsulation of cells into passively generated droplets follows a Poisson distribution, which is known to have a very low rate of single-cell encapsulation^9^. In contrast, active methods can generate droplets by actively applying external forces, providing greater control over droplet size and production frequency^10–13^. Although, electrical, magnetic, mechanical, optical, and acoustic forces have been used for actuating droplet generation^11–13^, the integration of external actuation in droplet-forming microfluidic platform inevitably adds complexity in device fabrication and instrumentation.

T-junction channel geometry has been widely used as a well-suited approach for both passive and on-demand continuous generation of droplets^14–24^. Typically, the aqueous phase enters the continuous oil phase from a side channel, forming dispersed droplets under specific flow conditions. In most of these systems, droplet formation is passive, relying on the interplay between shear and interfacial forces^16–24^. For example, the nascent aqueous droplet emerging from the side channel is ‘sheared-off’ by the advancing oil phase. Changing the flow rate ratio of two immiscible fluids modulates shear stress and droplet dimension. However, upstream flow rate adjustments are not reflected instantaneously at the T-junction due to flow resistance and compressibility of elements in many microfluidic systems, making this approach unsuitable for applications requiring demand-driven 'on' and 'off' droplet formation.

In one method for on-demand droplet formation, a pulse of pressure is applied to the water phase to generate a droplet at a T-junction^14,15^; this has the potential to control droplet generation at the individual droplet level. However, to do this requires knowledge of a pre-set threshold pressure for “switching off” droplet generation (i.e. inlet pressures which perfectly balance the forces between the two fluid phases at the T-junction), which is a trial-and-error process and prone to variations of droplet formation (e.g. due to surface modification)^14^. Recently, a dual-pressure-pulse driven droplet generation method, incorporated with feedback control based on real-time quantitative phase imaging, demonstrated good system response and low steady state error^25^. However, this complexity increases the barrier to integrating on-demand droplet generation with other modular operations in biological assays, such as cell sorting^7,26,27^. Currently, easy-to-use, on-demand droplet generation with excellent adaptability and practicability has yet to be achieved.

**Fig. 1:**
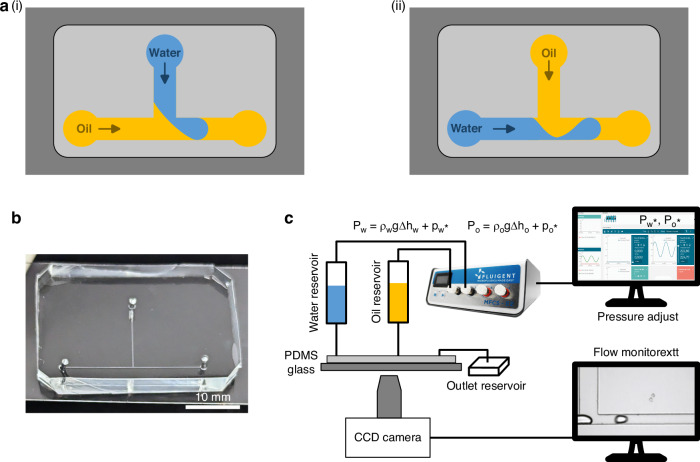
Pressure-Driven droplet formation at a microfluidic T-junction. **a** Schematic diagram of a T-junction microfluidic device used for droplet generation (not to scale). (i) example where water is in the side channel and the droplet is ‘sheared-off’ by oil flow; (ii) example where oil is in the side channel (this work) and the droplet is ‘cut-off’ by oil flow pressure increase after traversing the T-junction. **b** Photograph of the T-junction microfluidic device. **c** Schematic diagram of the experimental setup. A Fluigent pressure pump is used for pressure adjustment. The total pressures of oil (*P*_o_) and water (*P*_w_) at the oil and water reservoir are the sum of the hydrostatic pressure *ρ*_O_*g*∆*h*_O_ and *ρ*_W_*g*∆*h*_W_ and the pressure from the pressure pump ${P}_{w}^{* }$, ${P}_{o}^{* }$, respectively. Here, *ρ*, *g* and ∆*h* denote the density, gravitational acceleration and the height difference from the top of the liquid reservoir to the height of the microchip

Here, we propose a simple pressure-driven method for droplet generation at a T-junction, allowing robust on-demand operation. In contrast to the existing approaches (Fig. 1a(i)), where water is in the side channel, our method introduces *oil* in the side channel (Fig. 1a(ii)). Thus, a pressure-driven advance in the oil phase serves to ‘cut’ an aqueous stream passing along the ‘straight’ portion of the T-junction after the requisite volume for the droplet has traversed the T-junction.

Built upon experimental observations, we used classical Laplace–Young capillary pressure equations to develop an approximate and simple physical model to describe the mechanism underlying the droplet generation process, providing a guideline for microfluidic design and predicted pressure thresholds for on-demand droplet formation. We investigate whether this simplified approach, compared to more sophisticated and numerical simulations, effectively describes the droplet generation performance of T-junction devices across a range of oil and water pressures and device dimensions. Furthermore, we present a simple image-based method for rapid in situ optimisation of the working conditions for targeted on-demand droplet generation.

## Results and discussion

### Pressure-driven droplet generation at T-Junction

On-demand droplet generation requires precise control of the 'on' and 'off' states of droplet generation. Here, we introduce localised pressure-driven droplet formation at a T-junction, offering both flexibility and precise temporal control of droplet formation. In contrast to most previous systems^14–24^, we introduce the continuous oil phase from the side channel (Fig. 1), allowing precise control of droplet generation by applying and adjusting the pressure of the aqueous and/or the oil phases at the T-junction. With this configuration, droplet formation is primarily achieved by the pressure 'cutting-off' the leading water phase. Our approach leverages the fact that pressure changes propagate through the system almost instantaneously, offering a significant advantage in temporal control of droplet formation.

Comparison of the existing approach (Fig. 1a(i)) and our approach (Fig. 1a(ii)) shows that the oil/water menisci immediately prior to release of the formed droplet have different curvatures. In addition, after the droplet has been separated from the aqueous stream, under the conditions shown in Fig. 1a(i), the oil phase fully enters the aqueous side channel, whereas in our approach (Fig.1a(ii)), the aqueous stream remains partially occupying the junction region of the T-junction. The latter has significant implications for the pressure differentials required to achieve rapid on-demand droplet formation.

**Fig. 2:**
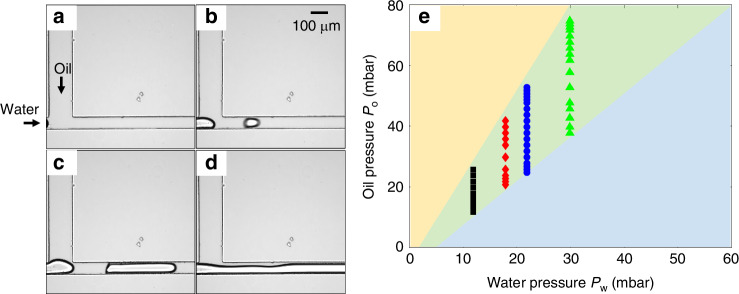
Droplet generation at different pressures of the oil and water phases. Water droplet generation at a constant water pressure of 11.9 mbar and different oil pressures: 30.3, 29.7, 15.7 and 9.7 mbar in **a**–**d**, respectively, for the microchannel with a channel height *h* = 75 µm, channel width *w*_o_ = 150 µm, *w*_w_ = 75 µm, and *w*_out_ = 75 µm, and channel length *l*_o_ = 10 mm, *l*_w_ = 10 mm and *l*_out_ = 1.5 mm for the water inlet, oil inlet, and outlet channels, respectively. The water enters the T-junction from the water channel on the left and is cut off into droplets by the oil flow from the top channel. Supplementary Videos 1-4 illustrate the four different formation regimes depicted in **a**–**d**. The mineral oil phase consists of 2 wt.% Span 80. **e** Pressure phase map for dispersed droplet generation. Droplets can be generated within the green region. Yellow and blue regions are for the conditions where oil pressure is higher than the upper threshold and lower than the lower threshold, respectively

Figure 1 shows the configuration of a T-junction device and the setup for droplet generation. The pressures of water and oil inlets are adjusted using a Fluigent pressure pump. Droplet generation at the T-junction is monitored by a CCD camera. By varying the combination of water and oil pressures, we observed different phenomena for droplet formation, as shown in Fig. 2 (Supplementary Videos 1–4). For a fixed water pressure, a high oil pressure causes the water phase to stop at the entrance of the T-junction, preventing droplet formation (Fig. 2a and Supplementary Video 1). When the oil pressure is reduced (Fig. 2b, c and Supplementary Videos 2, 3), the droplet starts to form, and its volume increases with the decrease of the oil pressure. With a further decrease in oil pressure, the water phase enters the outlet channel as a continuous flow, resulting in no droplet generation (Fig. 2d and Supplementary Video 4). The phase map for generating dispersed droplets (Fig. 2e) under these conditions clearly shows that the droplet generation region is sandwiched between two pressure thresholds. To generate dispersed droplets, moderate oil pressures are required for a given water pressure and vice versa; this also indicates that the thresholds of the dispersed droplet generation region can be utilised for the on-demand generation of droplets.

### Model for droplet generation

The ability to predict threshold conditions for on-demand droplet formation in T-junction devices allows active and automated control of droplet formation at the individual droplet level, greatly enhancing practicability and reliability. Based on experimental observations (Supplementary Videos 1–4 and Fig. 2), we can develop a physical model by analysing the critical stages of droplet formation. As illustrated above (Fig. 2e), two conditions should be met for dispersed droplets to be generated. For a given water pressure, oil pressure must be smaller than the upper threshold (beyond which the oil pressure is too high for the water to enter the T-junction) and greater than the lower threshold required for the oil phase to interrupt continuous water flow. Dispersed droplets can be generated at oil pressures between these two critical thresholds. Estimating values for these two thresholds will be discussed in the following sections.

### Conditions for the water phase to enter the T-Junction

As described in the introduction section, it may be that after release of a droplet, the aqueous stream is left partially occupying the junction region of the T-junction with the oil/water interface essentially static or moving. Alternatively, if the on-demand protocol dictates, the oil pressure might be allowed to increase, so forcing the oil/water interface to retreat into the aqueous channel. For this latter case, if the interface is static, the relationship between pressure across the meniscus and the oil/water contact angles at the PDMS channel walls is given by the Laplace–Young equation.

If the contact angle is ${\theta }_{1}$ at each of the four walls and the width and height are given by ${w}_{w}$ and $h$, when the oil/water interface is far from the side channel in the T-junction, the capillary pressure (or pressure difference between the oil and water streams in the vicinity of the junction), $\Delta {P}_{1}$, is given by Eq. 1a.

1a $$\Delta {P}_{1}=\gamma \left(-\frac{\cos {\theta }_{1}}{{w}_{w}}-\frac{\cos {\theta }_{1}}{{w}_{w}}-\frac{\cos {\theta }_{1}}{h}-\frac{\cos {\theta }_{1}}{h}\right)$$

**Fig. 3:**
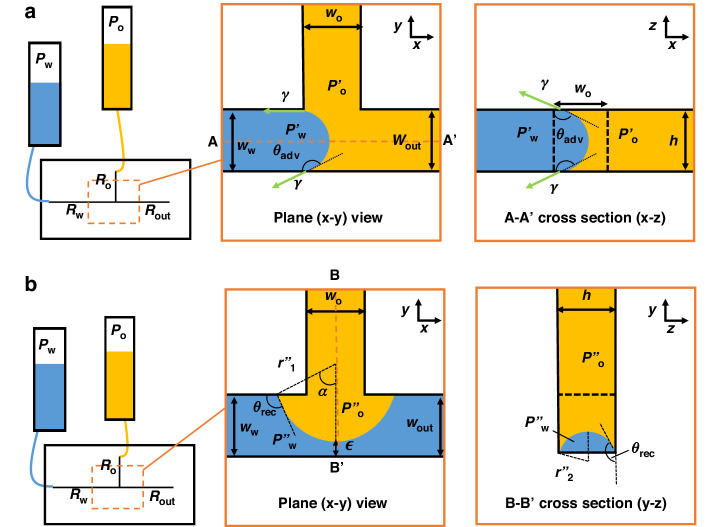
Schematic diagram of the critical conditions for droplet generation. **a** When the water phase entres the T-junction, shown in side and bottom views. **b** When the oil phase cuts off the water phase, shown in side and front views. *P*_o_, *P*_w_ are the pressures of oil (o) and water (w) at the reservoirs, while *P*_o_^′^ and *P*_w_^′^ in **a** and *P*_o_^′′^ and *P*_w_^′′^ in **b** are the oil and water pressures near the T-junction. *w*_o_, *w*_w_, *w*_out_ and *h* represent the widths of the oil inlet channel, water inlet channel, outlet channel and channel height at the T-junction, respectively. *R*_o_, *R*_w_ and *R*_out_ denote the hydraulic resistance of the whole oil channel, water channel and outlet channel, respectively. The resistances of all connecting tubes are considered. The pressure across the interface is $\varDelta {P}_{1}={P}_{w}^{{\prime} }-{P}_{o}^{{\prime} }$. The dashed line in the bottom view in **a** and the cross-section view in **b** indicate the location of the oil channel

For droplet-on-demand generation, the water pressure can be slowly increased until part of the oil/water meniscus reaches the PDMS boundary wall of the oil side channel (as shown in Fig. 3a). For the water droplet to move further into the junction region, a small pressure increase is needed. Experimentally this was observed, as shown in Supplementary Fig. S1. In particularly, close to the junction, the radius of curvature of the leading edge of the droplet approaches the width of the water channel ${w}_{w}$ (Supplementary Fig. S1, fitted circle), and the leading edge of the droplet becomes slightly separated from the water channel’s PDMS wall by a thin layer of oil originating from the oil channel. Thus, an upper limit for the water/oil pressure difference that may be required for the droplet to enter the junction region is given by setting ${\theta }_{1}$ at the water channel and the oil side channel interface equal to $\pi$ in Eq. 1a:

1b $$\Delta {P}_{1}=\gamma \left(-\frac{\cos \pi }{{w}_{w}}-\frac{\cos {\theta }_{1}}{{w}_{w}}-\frac{2\cos {\theta }_{1}}{h}\right)$$

Where ∆*P*_1_, the pressure across the interface when water is about to enter the junction, equals ${P}_{{\rm{w}}}^{{\prime} }-{P}_{{\rm{o}}}^{{\prime} }$ in Fig. 3a.

It should be noted that Eq. 1b represents an upper limit for ∆*P*_1_ close to the T-junction that arises when the oil phase has primed and completely wetted the channel prior to the formation of the aqueous droplet, thereby preventing the approaching water phase from immediately contact the wall directly in this area. However, when the water droplet enters the junction area, it partially moves into the oil side channel, leading to an increase in radius of curvature and consequent reduction in pressure ∆*P*_1_ required to keep the interface at a static position.

In recognition of the fact that the water channel width and channel height both influence the pressure required for on-demand generation of droplets with specified volumes, equations for optimising microfluidic designs can be developed accordingly. By introducing the aspect ratio *m*_w_ = *w*_w_*/h*, the expression of pressure difference across the oil/water interface from Eq. 1b is:

2 $$\varDelta {P}_{1}=\left(\frac{1-\cos {\theta }_{1}}{{m}_{w}}-2\cos {\theta }_{1}\right)\frac{\gamma }{h}$$

Assuming that before the water enters the T-junction, the velocity of the water phase is effectively zero (i.e. the on-demand droplet is waiting to be dispensed), the effect of water channel hydraulic resistance is small, and ${P}_{w}^{{\prime} }={P}_{w}$, where *P*_w_ is the pressure at the water inlets. However, *P*_o_^′^ is smaller than *P*_o_ (the oil inlet pressure) due to the pressure drop along the oil channel as a consequence of the non-zero oil velocity. For the threshold at the junction, we have:

3 $${P}_{w}^{{\prime} }={P}_{o}^{{\prime} }+\Delta {P}_{1}$$

To calculate the changes in inlet water or oil pressures required to dispense a droplet, we take the pressure at the exit of the outlet channel as the reference pressure (*P*_∞_ = 0), and denote the hydraulic resistance for the oil, water and outlet channels as *R*_o_, *R*_w_ and *R*_out_. Here, the value of *R*_out_ will depend on how much of each phase has entered the outlet channel. For many practical applications, the microfluidic structure is designed such that, in steady state operation, either a constant ratio of oil and water segments occupies the outlet channel, or the outlet channel’s width profile and length are adjusted so that only the region close to the T-junction contributes significantly to the outlet hydraulic resistance.

Thus, when the water channel is fully blocked, only oil flows in the outlet channel and the relation between inlet and near interface oil pressure is given by:

4 $${P}_{o}={P}_{o}^{{\prime} }/\left(1-\frac{{R}_{o}}{{R}_{o}+{R}_{{out}\left(o\right)}}\right)$$

Given $\Delta {P}_{1}={P}_{w}-{P}_{o}^{{\prime} }$, for the threshold when water is about to enter the junction, we have a linear relationship between the critical value for oil pressure at a given water i.e. the upper threshold *P*_o,max_ that would prevent droplet generation is:

5 $${P}_{o{\rm{\_}}\max }={C}_{1}{P}_{w}+{C}_{2}$$

where ${C}_{1}=({R}_{o}+{R}_{{out}\left(o\right)})/{R}_{{out}\left(o\right)}$, and ${C}_{2}=-({R}_{o}+{R}_{{out}\left(o\right)})/{R}_{{out}\left(o\right)}\varDelta {P}_{1}$. From Eq. 20 in the Material and methods section, $R=\frac{12\mu l}{{h}^{4}}A(m)$, where m is the aspect ratio and m = w/h. This gives ${C}_{1}=\frac{{l}_{o}A\left({m}_{o}\right)}{{l}_{{out}}A\left({m}_{{out}}\right)}+1$, where ${l}_{o}$ and ${l}_{{out}}$ are the corresponding pre-factors in the hydraulic resistance calculation of Eq. 20. Thus, the slope of the upper threshold oil pressure versus the water pressure reflects the ratio of the combined hydraulic resistance of the oil and outlet channels to the hydraulic resistance of the oil-filled outlet channel alone.

Finally, if the outlet channel is filled with oil, at water pressures below ${P}_{w}=\varDelta {P}_{1}$ (where $\varDelta {P}_{1}$ is given by Eq. 1), water cannot enter the T-junction occupied by the oil regardless of the oil pressure.

### Conditions for the oil phase to cut off the water phase

To generate dispersed droplets, the oil phase must interrupt the water flow as shown in Fig. 3b. When the water flows continuously from the water channel to the outlet channel passing through the T-junction, the water flow appears to block the oil channel completely (Supplementary Video 5), leading to reduced oil flow. This causes an increase in oil pressure at the junction, and so the interface moves down towards the T-junction channel side-wall next to the B’ label in the plane and cross-section views of Fig. 3b. Assuming the whole process is in a quasi-equilibrium state, therefore, the receding contact angle of the water/oil interface on the PDMS wall is *θ*_2_. To match the quasi-equilibrium state provided by the Laplace pressure at the convex, as shown in Fig. 3b the pressure across the interface, ∆*P*_2_ is approximated by:

6 $$\Delta {P}_{2}=\gamma \left(\frac{1}{{r}_{1}^{{\prime} {\prime} }}-\frac{1}{{r}_{2}^{{\prime} {\prime} }}\right)={P}_{o}^{{\prime} {\prime} }-{P}_{w}^{{\prime} {\prime} },$$

where *P*_o_^′′^ and *P*_w_^′′^ are the pressures at the junction across the oil/water interface when the oil phase protrudes from the T-junction side channel so as to ‘cut’ off the water droplet.

Assuming the velocity of the oil phase is close to zero, i.e. the water phase is substantially impeding the oil’s emergence from the oil channel. This leads to ${P}_{o}^{{\prime} {\prime} }={P}_{o}$, and $\Delta {P}_{2}={P}_{o}-{P}_{w}^{{\prime} {\prime} }$ in Fig. 3b. If there is a slight oil flow velocity, the water/oil interface in the T-junction that is coaxial with the oil side channel structure moves forward until the oil touches the PDMS channel side-wall that forms the top of the ‘T’ in the T-junction (i.e. the wall closest to B’ in the cross section view in Fig. 3). When this happens, the contact angle at this wall suddenly becomes much smaller than *θ*_2_ due to the oleophilic and hydrophobic properties of PDMS, allowing the water/oil interface to completely move towards this side-wall of the outlet channel, without further increase of oil pressure. During this droplet release stage, the water adjacent to the side-wall forms a thin filament quickly which is easily be broken with the help of the shear stress from the oil flow, detaching the droplet from the aqueous stream. Therefore, the moment when the interface arrives at the bottom channel is defined as the critical condition for oil to cut off the water phase. And

7 $${r}_{2}^{{\prime} {\prime} }=\frac{h}{-2\cos {\theta }_{2}}$$

where *h* is the height of the channel. The height of the arc in the B-B’ cross-section view of Fig. 3b is ${\epsilon }_{\min }=\frac{h(1-\sin {\theta }_{2})}{-2\cos {\theta }_{2}}$. In the plane view of Fig. 3b, when ${w}_{o} \,<\, 2{r}_{1}^{{\prime} {\prime} }\sin {\theta }_{2}$ and ${w}_{w}-{\epsilon }_{\min } > \frac{{w}_{o}}{2\sin {\theta }_{2}}(1+\cos {\theta }_{2})$, the oil/water interface recedes, and the condition ${w}_{w}-{\epsilon }_{\min }={r}_{1}^{{\prime} {\prime} }(1+\cos {\theta }_{2})$ needs to be met. This leads to:

8 $${r}_{1}^{{\prime} {\prime} }=\frac{{w}_{w}-{r}_{2}^{{\prime} {\prime} }\left(1-\sin {\theta }_{2}\right)}{1+\cos {\theta }_{2}}$$

So

9 $$\Delta {P}_{2}=\left(\frac{2\cos {\theta }_{2}\left(\cos {\theta }_{2}+\sin {\theta }_{2}-2{m}_{w}\cos {\theta }_{2}\right)}{2{m}_{w}\cos {\theta }_{2}+1-\sin {\theta }_{2}}\right)\frac{\gamma }{h}$$

When the oil channel is fully blocked and before it cuts off the continuous water phase, $\frac{{P}_{w}-{P}_{w}^{{\prime} {\prime} }}{{P}_{w}-{P}_{\infty }}=\frac{{R}_{w}}{{R}_{w}+{R}_{{out}(w)}}$, where ${P}_{\infty }=0$. So ${P}_{W}^{{\prime} {\prime} }={R}_{{out}\left(w\right)}/\left({R}_{w}+{R}_{{out}\left(w\right)}\right){P}_{W}$. Given that $\Delta {P}_{2}={P}_{o}-{P}_{w}^{{\prime} {\prime} }$, which leads to:

10 $${P}_{o}^{{\prime} {\prime} }-\Delta {P}_{2}={P}_{w}^{{\prime} {\prime} }$$

The linear relationship between oil and water pressure for the lower boundary, *P*_o,min_, is given by (c.f. Equation 5):

11 $${P}_{{o\_}\min }={C}_{3}{P}_{w}+{C}_{4}$$

where ${C}_{3}={R}_{{out}\left(w\right)}/({R}_{w}+{R}_{{out}\left(w\right)})$ and ${C}_{4}=\varDelta {P}_{2}$. Since the PDMS channel wall is hydrophobic and oleophilic, there would always be oil near the corner of the outlet channel. Therefore, the oil channel is not strictly blocked in the case of a flat interface at the junction, and the local water pressure is underestimated due to the existence of oil in the outlet channel. To address this, we use a constant outlet resistance ${R}_{{out}}={R}_{{out}(o)}$.

### Experimental verification of critical pressure calculations in droplet generation

**Fig. 4:**
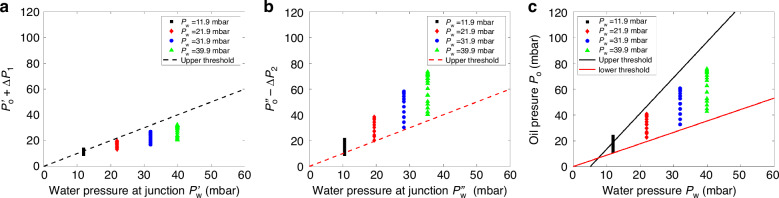
Threshold comparison between experimental observation and model results. Droplet generation map of **a**, **b** local pressures at the T-junction and **c** the overall inlet pressures. The parameters of the device are *h* = 32 µm, *w*_o_ = 54 µm, *w*_w_ = 44 µm, *w*_out_ = 104 µm, *l*_o_ = 0.2 mm, *l*_w_ = 0.2 mm, and *l*_out_ = 1.1 mm. Red and black dotted lines in **a**, **b** represent thresholds based on Eq. 3. and Eq. 10, respectively. Solid lines in **c** represent the thresholds based on Eq. 5 & Eq. 11

Figure 4 compares experimental results with model predictions of the two thresholds for droplet generation. The threshold in Fig. 4a represents the critical upper pressure of oil at the junction for water to enter the T-junction based on Eq. 3. When the oil pressure exceeds this threshold, water is unable to enter the junction. Consequently, no droplets form. This behaviour indicates that the T-junction functions as a valve for droplet generation. At the T-junction, the interfacial tension between water and oil must be overcome by the pressure difference across the interface for a droplet to form. Below this critical threshold, water can enter the junction, and, as illustrated in Fig. 2, lower oil pressure results in larger droplet volumes.

To generate dispersed droplets, a second criterion must also be satisfied: the oil phase must cut off the water flow. This is demonstrated in Fig. 4b, where the dotted red line presents the relationship between the local pressure across the oil/water interface at the junction, as derived from Eq. 10. When the oil pressure is below this threshold, the oil flow can not cut off the continuous flow of water through the T-junction. To elucidate the combined contributions of these two criteria and their connection to the operating parameters (inlet water and oil pressures) for the microfluidic device, Fig. 4c shows the overall thresholds for water-in-oil droplet generation in a T-junction.

### Droplet volume

Accurate droplet volume is a critical factor in droplet generation. For continuous droplet generation, ref. ^16^ proposed a relationship between droplet length and the flow rate ratio:

12 $$\frac{L}{W}=\frac{{{\rm{Q}}}_{{\rm{w}}}}{{Q}_{o}}+1.$$

Under controlled pressure conditions, the equivalent flow rates must be derived for different pressures. For the water channel, ${Q}_{w}=({P}_{w}-{P}_{w}^{{\prime} })/{R}_{w}$, where ${P}_{w}^{{\prime} }={P}_{o}^{{\prime} }+\Delta {P}_{1}$, ${P}_{w}={P}_{o,\max }* {R}_{{out}\left(o\right)}/({R}_{{out}\left(o\right)}+{R}_{O})+\Delta {P}_{1}$. Simplifying,

$${Q}_{w}\cong \frac{{P}_{o,\max }* \frac{{R}_{{out}\left(o\right)}}{{R}_{{out}\left(o\right)}+{R}_{O}}-{P}_{o}^{{\prime} }}{{R}_{w}}=\frac{{P}_{o,\max }* \frac{{R}_{{out}\left(o\right)}}{{R}_{{out}\left(o\right)}+{R}_{O}}-\frac{{R}_{{out}\left(o\right)}}{{R}_{{out}\left(o\right)}+{R}_{O}}{P}_{o}}{{R}_{w}}$$

This reduces to:

13 $${Q}_{{\rm{w}}}\cong \frac{{{\rm{R}}}_{{\rm{out}}\left({\rm{o}}\right)}}{\left({{\rm{R}}}_{{\rm{out}}\left({\rm{o}}\right)}+{{\rm{R}}}_{{\rm{O}}}\right){{\rm{R}}}_{{\rm{w}}}}({{\rm{P}}}_{{\rm{o}},\max }-{{\rm{P}}}_{{\rm{o}}})$$

For the oil flow, the continuous oil phase has an average flow rate of ${Q}_{o}=({P}_{o}-{P}_{o}^{{\prime} })/{R}_{o}$ before water fully occupies the outlet channel. For a constant water pressure, we have ${P}_{\min }={P}_{w}^{{\prime} }+\Delta {P}_{2}={P}_{w}* {R}_{{out}(w)}/({R}_{{out}(w)}+{R}_{w})+\Delta {P}_{2}$. When ${Q}_{o}$ approaches to zero, ${P}_{o}^{{\prime} }={P}_{\min }$. Thus, for a given water pressure and small ${P}_{o}$:

14 $${Q}_{o}\cong ({P}_{o}-{P}_{\min })/{R}_{o}.$$

From Eqs. 13 & 14, the flow rate ratio can be expressed as:

15 $$\frac{{Q}_{{\rm{w}}}}{{Q}_{o}}={C}_{5}\frac{{R}_{{out}\left(o\right)}{R}_{o}}{\left({R}_{{out}\left(o\right)}+{R}_{O}\right){R}_{w}}\frac{{P}_{{\rm{o}},\max }-{P}_{{\rm{o}}}}{{P}_{o}-{P}_{\min }},$$

where ${C}_{5}$ is a factor accounting for deviations in pressure within the continuous phase, threshold conditions, and differences between controlled pressure and controlled flowrate conditions.

The relationship between droplet volume and length in the microchannel is given by^28^:

16 $$V=\frac{\pi }{4}{whL}-\frac{\pi }{12}w{h}^{2}$$

Thus, the volume of the droplet can be expressed as:

17 $$V=\frac{\pi }{4}{w}^{2}h\left(\frac{L}{w}-\frac{h}{3w}\right)=\frac{\pi }{4}{w}^{2}h\left({C}_{5}\frac{{R}_{{out}\left(o\right)}{R}_{o}}{\left({R}_{{out}\left(o\right)}+{R}_{O}\right){R}_{w}}\frac{{P}_{{\rm{o}},\max }-{P}_{{\rm{o}}}}{{P}_{o}-{P}_{\min }}+\frac{3w-h}{3w}\right)$$

Specially, the minimum droplet volume that the T-junction can produce is ${V}_{o}=\frac{\pi }{4}{wh}* \frac{3w-h}{3}$.

### Model evaluation and implementation

Droplet generation is influenced by many parameters, such as pressure, liquid physical properties and the geometry of the microchannels. To evaluate the performance of our model, we varied the T-junction geometries and the oil/water interfacial tension, comparing the model’s predictions with the experimental results.

#### Influence of device parameters

**Fig. 5:**
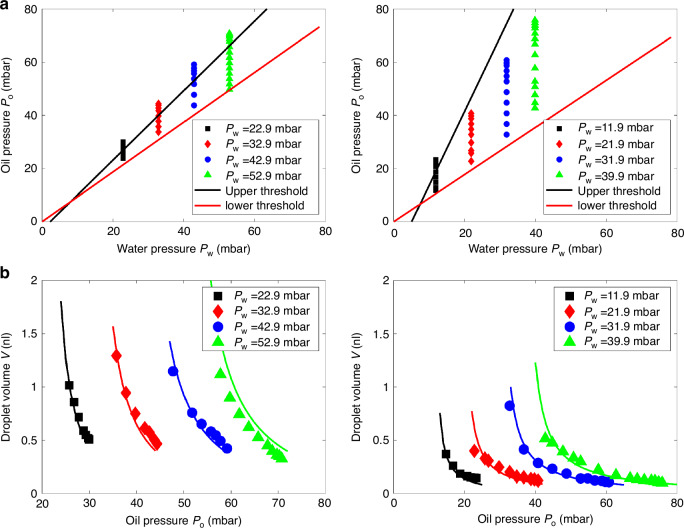
Model evaluation for different devices. **a** Pressure map and **b** droplet volume for droplet generation in two different devices: (i) *h* = 75 µm, *w*_o_ = 150 µm, *w*_w_ = 75 µm, *w*_out_ = 75 µm, *l*_o_ = 10 mm, *l*_w_ = 10 mm, and *l*_out_ = 1.5 mm. (ii) *h* = 32 µm, *w*_o_ = 54 µm, *w*_w_ = 44 µm, *w*_out_ = 104 µm, *l*_o_ = 0.2 mm, *l*_w_ = 0.2 mm, and *l*_out_ = 1.1 mm. Black and red solid lines represent thresholds based on Eq. 5 and Eq. 11 in the model. The fitting parameters ${C}_{5}$ is 0.5 and 0.08 for devices (i) and (ii), respectively

The model can estimate pressure thresholds for a given device geometry, as shown in Fig. 5a. For the device (ii) in Fig. 5a, where the outlet channel is larger than the water channel, droplet generation becomes easier, allowing a broader pressure range to control droplet volume compared to the device (i). As shown in Fig. 5b, for a fixed water pressure, droplet volume decreases as oil pressure increases. By setting the fitting parameter ${C}_{5}$ in Eq. 17 to ${C}_{5}=0.08$ *and* $0.5$, for device (i) and (ii), respectively, the measured droplet volume aligns closely with the model’s predictions, as seen in Fig. 5b. The droplet volume reaches a minimum value regardless of changes in water pressure, indicating that the droplet size is constrained by the dimensions of the microfluidic channels in the T-junction. To produce droplets with targeted volumes, the microfluidic device must therefore be designed with appropriate geometrical considerations.

#### Influence of interfacial tension

**Fig. 6:**
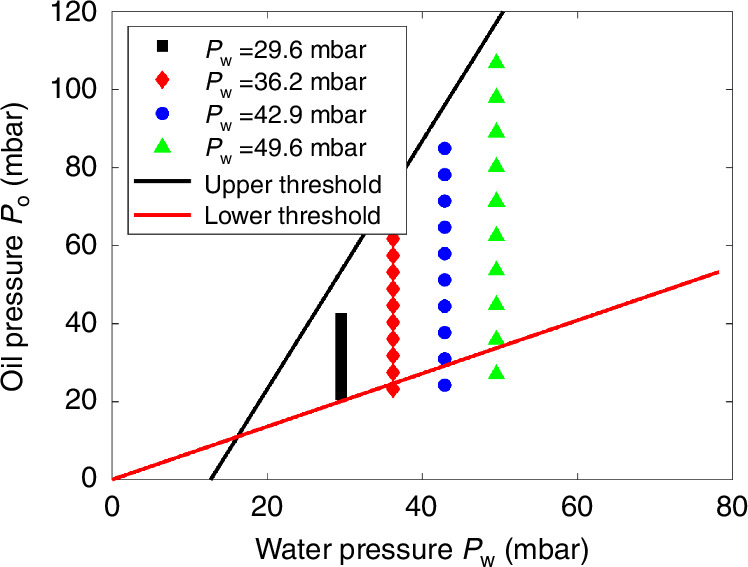
Pressure map for droplet generation for the oil phase of mineral oil mixed with 2 wt.% Span 80 as a surfactant. The device used is the same as in Fig. 5(i). The interfacial tension between pure mineral oil and water is 24–5 mN/m^28^. The black and red solid lines indicate the upper and lower thresholds from the model, respectively

Surface tension significantly affects droplet generation, and surfactant is widely used to reduce the water/oil interfacial tension. Figure 6 shows that adding a surfactant to the oil phase (2 wt% Span 80) extends the region for droplet generation compared to conditions without surfactant (i.e. Fig. 5a(i)). The interfacial tension maintains the boundary between the oil and water phases, hindering droplet formation. By reducing interfacial tension, surfactants lower the pressure difference required across the interface, thereby expanding the range of operating pressures for droplet generation. This demonstrates that reducing interfacial tension facilitates droplet generation.

#### Model implementation for on-demand droplet formation

**Fig. 7:**
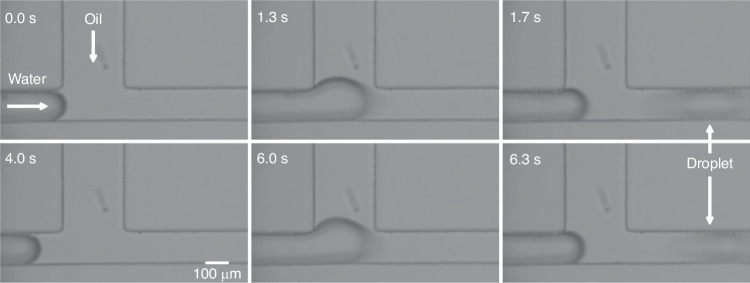
Time series of representative images of on-demand droplet generation at a pre-defined ~5 s interval. The process for this interval and others can be found in Supplementary Video 2. The device and condition used are the same as in Fig. 6. The water pressure was set to 20.0 mbar, with the 'off' state oil pressure at 23.7 mbar and the 'on' state oil pressure at 21.0 mbar

With the predicted pressure thresholds, pre-setting conditions for on-demand droplet formation can be easily achieved. A defined range of pressures suitable for either the 'on' state or the 'off' state of droplet generation (e.g. the green region and yellow region in Fig. 2c, respectively) significantly improves operational robustness. To demonstrate this, the oil inlet was connected via a valve to two oil reservoirs, enabling the pressure at the oil inlet to be switched between the 'on' and 'off' states. Figure 7 and Supplementary Video 6 show the individual water-in-oil droplets were reliably created with well-defined intervals between successive droplets. This precision, combined with the simplicity of controlling individual droplet formation, highlights the significant benefits of utilising model predictions for guiding droplet generation.

### Solutions to overcome parameter uncertainties

Our model provides a good guideline for designing droplet-on-demand microfluidic devices and determining the operating range of pressure for droplet generation. However, surface treatment of the devices and the customised use of surfactants can introduce uncertainties to the nominal parameters used with the model, compromising the accuracy of predictions. To address this, we developed a useful in situ method to facilitate the determination of threshold pressures based on droplet generation frequency, as described below.

**Fig. 8:**
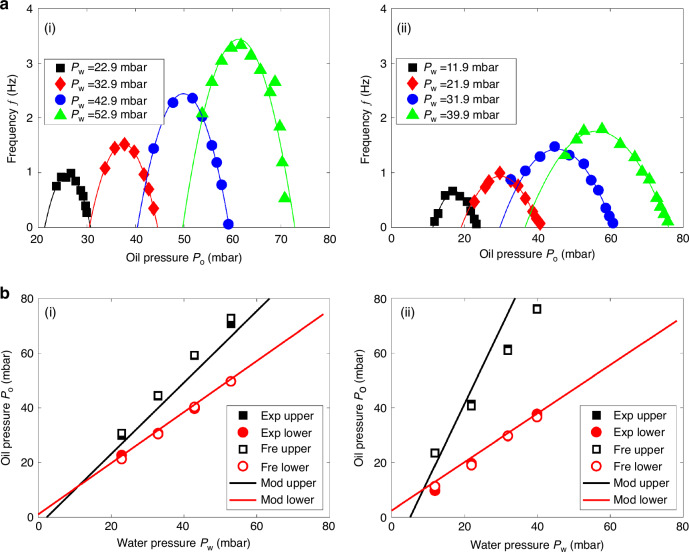
Solution to overcome parameter uncertainties via frequency monitoring. **a** Frequency of droplet generation as a function of *P*_o_ and **b** thresholds of droplet generation as a function of *P*_w_ for two different devices: (i) *h* = 75 µm, *w*_o_ = 150 µm, *w*_w_ = 75 µm, *w*_out_ = 75 µm, *l*_o_ = 10 mm, *l*_w_ = 10 mm, and *l*_out_ = 1.5 mm. (ii) *h* = 32 µm, *w*_o_ = 54 µm, *w*_w_ = 44 µm, *w*_out_ = 104 µm, *l*_o_ = 0.2 mm, *l*_w_ = 0.2 mm, and *l*_out_ = 1.1 mm. The solid line shows the parabolic fitting for each case. Supplementary Video 7 illustrates the droplet generation frequency at various oil pressures, with a constant water pressure (*Pw*) maintained at 11.9 mbar

We observed a parabolic relation between the droplet generation frequency, *f*, and the oil pressure *P*_o_ for a given water pressure *P*_w_, as shown in Fig. 8a and Supplementary Video 7. When the oil pressure increases and reaches the maximum oil pressure *P*_o,max_, the water phase can no longer enter the junction. At this point, ${P}_{w}={P}_{w}^{{\prime} }=P{o}^{{\prime} }+\Delta {P}_{1}=Po,\max \cdot \frac{{R}_{{out}\left(o\right)}}{{R}_{{out}\left(o\right)}+{R}_{o}}+\Delta {P}_{1}.$ Conversely, when the oil pressure is too low, the oil phase can no longer cut off the water phase. The minimum oil pressure *P*_*o,min*_ can be approximately expressed as: ${Po},\min \cong {P}_{w}^{{\prime} }+\Delta {P}_{2}={P}_{w}\cdot \frac{{R}_{{out}\left(w\right)}}{{R}_{{out}\left(w\right)}+{R}_{w}}+\Delta {P}_{2}$_._ Thus, *P*_o,max_ and *P*_o,min_ are independent of *P*_o_ but are functions of *P*_w_.

This finding allowed us to introduce a practical method for determining the thresholds in situ, particularly when the surfactant parameters are unavailable. First, the frequency of droplet generation at a specific range of pressures is recorded, and a parabolic relation is fitted to the data. Using the relation, the two oil pressure values at which the frequency equals zero can be calculated—these correspond to the upper and lower pressure thresholds for droplet generation at a given water pressure. To validate the idea, the thresholds derived using this approach were compared with those measured experimentally, and the results, shown in Fig. 8b, demonstrate good agreement, supporting the reliability of this practical method.

## Conclusion

We developed a simple method for pressure-driven, on-demand droplet formation at a T-junction with precise temporal control over individual droplet formation. Based on experimental observations, we proposed and validated a physical model describing critical stages of droplet formation and predicting pressure thresholds for switching droplet generation on and off. The model effectively correlates device geometry, surface properties, interfacial tension, and operational pressures with droplet formation, offering valuable guidance for the systematic design of devices and droplet formation.

To address uncertainties associated with nominal parameters, such as surface tension, arising from real-world complexity, we introduced a simple in situ optimisation method based on droplet generation frequency to determine threshold conditions. Our findings offer valuable guidelines for the design and automation of robust droplet-on-demand microfluidic systems, which can be readily implemented in conventional laboratories for a broad range of applications, such as manipulating individual droplets for cell encapsulation and cell sorting.

## Materials and methods

### Chemicals

Mineral oil (Sigma-Aldrich, No. 330779) and distilled water (Invitrogen UltraPure, Fisher Scientific, No. 11538646) were used as the continuous and dispersed phases for droplet generation. Surfactant Span 80 (Sigma-Aldrich, No. S6760) was used to adjust the interfacial tension between oil and water. Details on the influence of Span 80 concentration on interfacial tension can be found in ref. ^29^.

### Fabrication of microfluidic devices

The microfluidic devices were fabricated via soft lithography. In brief, the design of the microchannel was transferred via photolithography from a mask onto a 4-inch silicon wafer spin-coated with photoresist SU-8 and pre-baked. Subsequently, the wafer was post-baked and developed, creating a mould for polydimethylsiloxane (PDMS) casting. After pre-baking, the wafer was post-baked and developed, creating a mould for polydimethylsiloxane (PDMS) casting.

A mixture of PDMS oligomer and curing agent (10:1 ratio) was poured onto the mould and degassed in a vacuum chamber. The mould with PDMS was then baked in an oven at 70 °C for 3–4 h. The cured PDMS was cut from the mould, punched with through-holes for silicone tube connections, cleaned, and bonded to pre-cleaned glass slides (Knittel Glass, No. 3 thickness, MS0088) using oxygen plasma treatment. Silicone tubes connected the PDMS device’s inlet channels to the water and oil reservoirs, while the outlet channel and reservoir surfaces remained open to the atmosphere.

Hydrostatic pressure at the channel inlets was controlled by adjusting the heights of the reservoirs, with precise pressure control provided by Fluigent pumps (MCF-EZ).

### Optical microscopy

Droplet formation was recorded using a Zeiss Observer A1 inverted microscope, a Thorcam DCC1545M CMOS camera for high-speed video recording.

### Channel hydraulic resistance

The hydraulic resistance can be calculated using the following equation:

18 $$Q=\frac{\Delta P}{R}$$

where ∆*P* is the pressure drop in the channel, and *Q* is the flow rate of the continuous flow. For a rectangular channel, the flow rate is given by (Eq. 2.48 d in ref. ^23^):

19 $$Q=\frac{h{w}^{3}\varDelta P}{12\mu l}\left[1-\frac{192w}{{\pi }^{5}h}\mathop{\sum }\limits_{i=1}^{\infty }\frac{\tanh \frac{(2i-1)\pi h}{2w}}{{(2i-1)}^{5}}\right]$$

where *h*, *w* and *l* are the height, width and length of the channel, *µ* is the viscosity of the fluid. It is noteworthy that the definition of *h* and *w* here are different from those in ref. ^30^.

Therefore, the hydraulic resistance can then be expressed as:

20 $$R=\frac{12\mu l}{{h}^{4}}A(m)$$

where m = w/h is the aspect ratio, and $A\left(m\right)={m}^{-3}{\left[1-\frac{192m}{{\pi }^{5}}\mathop{\sum }\limits_{i=1}^{\infty }\frac{\tanh \frac{\left(2i-1\right)\pi }{2m}}{{\left(2i-1\right)}^{5}}\right]}^{-1}$.

To simplify *A*(*m*), a polynomial fit is applied, truncating the series to *n* = 4:

21 $$A\left(m\right)=\left\{\begin{array}{l}{(-0.1454{m}^{3}+0.7475{m}^{2}-0.1968m+0.0165)}^{-1}\\ {(1.0019m-0.5802)}^{-1}\end{array}\right.\begin{array}{l}0.3\le m\le 1\\ 1\le m\le 10\end{array}$$

## Supplementary information

Supplementary Video 1 (Fig. 2a)

Supplementary Video 2 (Fig. 2b)

Supplementary Video 3 (Fig. 2c)

Supplementary Video 4 (Fig. 2d)

Supplementary Video 5 cut_off

Supplementary Video 6 on-demand droplet generation

Supplementary Video 7 pressure-frequency

Supplementary information
