# Supplementary material for: On-demand droplet formation at a T-junction: modelling and validation
Source: Microsyst Nanoeng. 2025 May 19;11:94. doi: 10.1038/s41378-025-00950-2 (PMC12086211; doi:10.1038/s41378-025-00950-2)
Supplement: Supplementary file 8 — Supplementary information [file 41378_2025_950_MOESM8_ESM.docx]

**On-Demand Droplet formation at a T-junction: modelling and validation**

Hongyu Zhao^1,2^, William Mills^1^, Andrew Glidle^1^, Peng Liang^3,4^,

Bei Li^3,4^, Jonathan M. Cooper^1^, Huabing Yin^1*^

^1*^Division of Biomedical Engineering, James Watt School of Engineering, University of Glasgow, Glasgow, G12 8LT, Scotland, UK.

^2^Institute for Multiscale Thermofluids, School of Engineering, the University of Edinburgh, Edinburgh EH9 3BF, Scotland, UK.

^3^HOOKE Instruments Ltd., NO. 77 Yingkou Road, Changchun, 130033, Jilin, China.

^4^Changchun Institute of Optics, Fine Mechanics and Physics, Chinese Academy of Sciences, Changchun, 130033, Jilin, China.

*Corresponding author. E-mail(s): [huabing.yin@glasgow.ac.uk](mailto:huabing.yin@glasgow.ac.uk)

Supplementary Information


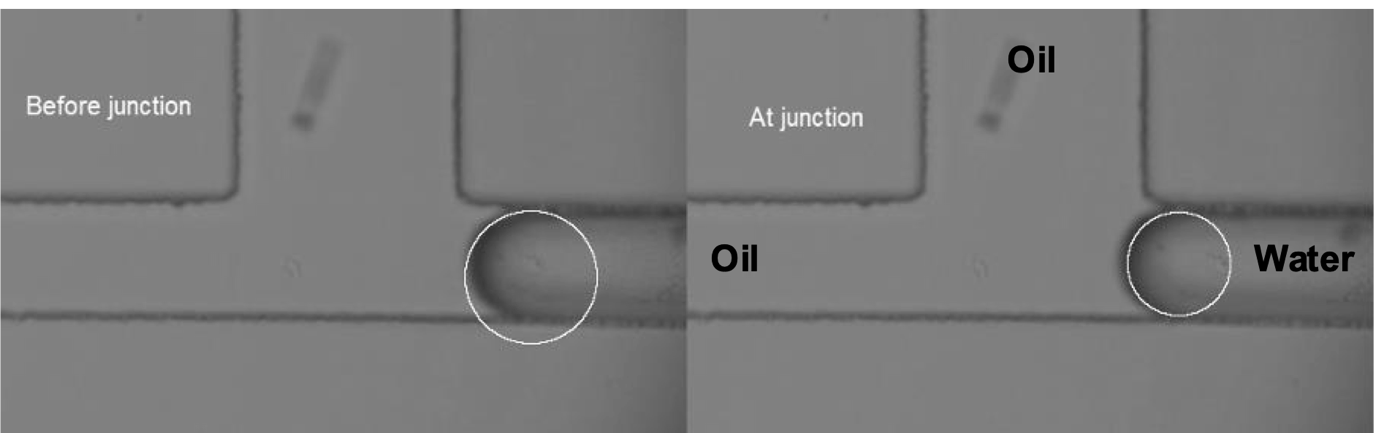


**Supplementary Figure S1**. Optical images showing different curvatures of the leading edge of the water/oil meniscus at various positions. The white circles fit the leading edge of droplet at the contact point between the water meniscus and the upper channel wall. The slightly different diameters of the white circles indicate differences incurvature.


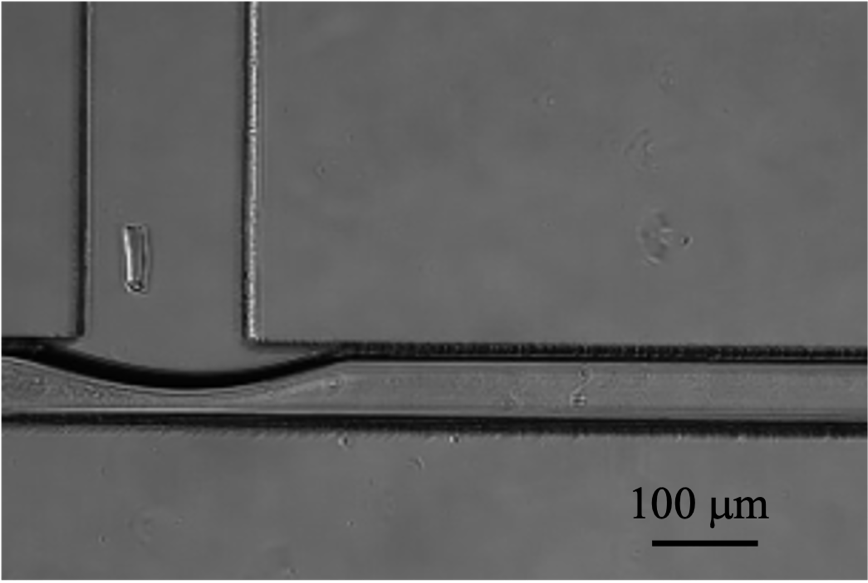


**Supplementary Figure S2**. Optical image of the water-oil interface during droplet formation, illustrating pressure-driven "cutting" of the water phase.
